# Early psychological impact of the 2019 coronavirus disease (COVID-19) pandemic and lockdown in a large Spanish sample

**DOI:** 10.7189/jogh.10.020505

**Published:** 2020-12

**Authors:** Leticia García-Álvarez, Lorena de la Fuente-Tomás, María Paz García-Portilla, Pilar A Sáiz, Carlota Moya Lacasa, Francesco Dal Santo, Leticia González-Blanco, María Teresa Bobes-Bascarán, Mercedes Valtueña García, Clara Álvarez Vázquez, Ángela Velasco Iglesias, Clara Martínez Cao, Ainoa García Fernández, María Teresa Bascarán Fernández, Almudena Portilla Fernández, Julia Rodríguez Revuelta, Elisa Seijo Zazo, Paula Zurrón Madera, María Suárez Álvarez, Ángeles Paredes Sánchez, Claudia Fernández Delgado, Silvia Casaprima Suárez, Isabel Menéndez Miranda, Luis Jiménez Treviño, Gonzalo Paniagua Calzón, Iciar Abad, Cristina Pedrosa Duque, Leonor Riera, Pedro Marina González, Eduardo Fonseca Pedrero, Julio Bobes

**Affiliations:** 1 Department of Psychiatry, Universidad de Oviedo, Oviedo, Spain; 2 Centro de Investigación Biomédica en Red de Salud Mental (CIBERSAM), Spain; 3 Instituto de Investigación Sanitaria del Principado de Asturias (ISPA), Oviedo, Spain; 4 Instituto Universitario de Neurociencias del Principado de Asturias (INEUROPA), Oviedo, Spain; 5 Department of Psychology, Universidad de Oviedo, Oviedo, Spain; 6 Servicio de Salud del Principado de Asturias (SESPA) Oviedo, Spain; 7 Department of Educational Sciences, Universidad de la Rioja, Spain

## Abstract

**Background:**

Epidemic outbreaks have significant impact on psychological well-being, increasing psychiatric morbidity among the population. We aimed to describe the early psychological impact of COVID-19 and its contributing factors in a large Spanish sample, globally and according to mental status (never mental disorder NMD, past mental disorder PMD, current mental disorder CMD).

**Methods:**

An online questionnaire was conducted between 19 and 26 March, five days after the official declaration of alarm and the lockdown order. Data included sociodemographic and clinical information and the DASS-21 and IES questionnaires. We analysed 21 207 responses using the appropriate descriptive and univariate tests as well as binary logistic regression to identify psychological risk and protective factors.

**Results:**

We found a statistically significant gradient in the psychological impact experienced in five domains according to mental status, with the NMD group being the least affected and the CMD group being the most affected. In the three groups, the depressive response was the most prevalent (NMD = 40.9%, PMD = 51.9%, CMD = 74.4%, *F* = 1011.459, *P* < 0.001). Risk factors were female sex and classification as a case in any psychological domain. Protective factors were younger age and ability to enjoy free time. Variables related to COVID-19 had almost no impact except for having COVID-19 symptoms, which was a risk factor for anxiety in all three groups.

**Conclusions:**

Our results can help develop coping strategies addressing modifiable risk and protective factors for each mental status for early implementation in future outbreaks.

The coronavirus disease (COVID-19) pandemic threatens public health worldwide [1]. In December 2019, an outbreak of severe pneumonia of unknown aetiology was identified in the city of Wuhan, China [2]. The first coronavirus case in Europe was reported in Germany in late January, and days later, related to that first case, the first patient infected with COVID-19 was identified in Spain. Since then, the number of cases has been increasing exponentially, spreading throughout the entire country (**Figure 1**).

**Figure 1:**
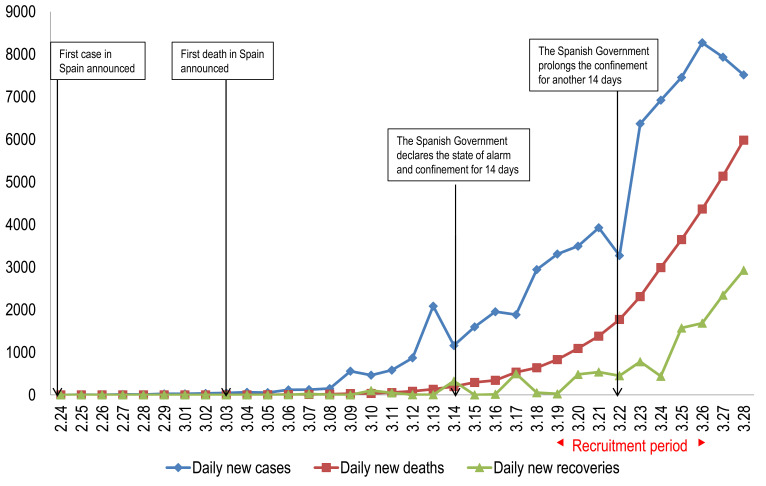
The national epidemic trend of the outbreak of the coronavirus disease 2019 (COVID-19) and socio-psychological milestones in Spain from February 24 to March 28, 2020.

Previous studies have shown that epidemic outbreaks have significant impact on mental health and psychological well-being [3,4], increasing psychiatric morbidity among population [5]. As Khan and Huremovic [6] point out two parallel processes occur in mental health at different levels in pandemic outbreaks. In China, the COVID-19 outbreak has caused emotional distress and had a negative impact on mental health [7]. During the initial phase of the epidemic, more than half of the general population reported a strong psychological impact and approximately a third reported moderate to severe anxiety [8].

Furthermore, there is evidence that the psychological effects of infection outbreaks can be felt months or even years later [9,10]. Three years after the Severe Acute Respiratory Syndrome (SARS) outbreak in China, 23% of health workers still reported moderate to severe depressive symptoms [10]. A better understanding of the psychological impact of the COVID-19 outbreak is crucial to design coping programs that may mitigate these responses during such outbreaks [11,12].

To our knowledge, there has been a paucity of studies examining the impact of COVID-19 on the mental health of the general [8], although some papers have been published on the expected consequences in countries such as Italy and Japan [13,14]. The objective of this study is to describe the early impact of the COVID-19 pandemic and lockdown situation on mental health and explore the factors that may contribute to or mitigate that impact in a large sample of the Spanish population. Furthermore, we also aim to compare the psychological impact according to absence or presence of past / current mental disorders. We hypothesize that the COVID-19 pandemic and lockdown will cause greater distress in individuals with current or past psychiatry disorders.

## METHODS

### Design

This is a cross-sectional anonymous online survey conducted between 14:30 on 19 March and 16:30 on 26 March, five days after the official declaration of alarm.

The recruitment strategy was a virtual snowball sampling that, although its limitations, it may be appropriate in the case of hard to reach populations [15], like this situation (state of alarm and lockdown). The behavior of the Spanish population in response to the current situation is not known [15]. To improve the external validity, we used the respondent-driven sampling method [16] to select subjects based on the researchers' interpersonal connections through social networks and the email to contact to different population profiles and organizations. We encouraged participation and maximum dissemination of the survey.

### Ethics

The study was conducted according to the ethical principles of the Declaration of Helsinki [17]. The Clinical Research Ethics Committee of Hospital Universitario Central de Asturias in Oviedo approved the study protocol (Ref. 2020.162) on 16 March, and online informed consent was obtained from all participants before enrolment.

### Participants

Of a total of 21 279 Internet surveys obtained, 72 were excluded for failure to meet the minimum age criterion. Inclusion criteria were 1)>17 years of age and 2) providing informed consent to participate in the study by clicking “I am of legal age and wish to participate in this project”. Exclusion criteria were designed to be minimal in order to obtain a representative sample of the general population and consisted of being under 18 years of age or refusing to participate.

There was an over-representation of the population of the Principality of Asturias and Cantabria. Catalonia and the Valencian Community were underrepresented, but the proportion of respondents from Madrid (the most affected region) was closer to the real one (**Table 1**).

**Table 1:** Geographical distribution of the Spanish and study populations.

	Spanish population		Study sample	
	**N**	**%**	**%**	**n**
**Total**	47 026 208			21207
Andalusia	8 414 240	17.9	8.4	1979
Aragon	1 319 291	2.8	2.3	263
Asturias, Principality of	1 022 800	2.2	36.2	7682
Balearic Islands	1 149 460	2.4	0.8	169
Canary Islands	2 153 389	4.6	1.4	298
Cantabria	581 078	1.2	11.9	2529
Castile and León	2 399 548	5.1	4.0	876
Castilla - La Mancha	2 032 863	4.32	1.4	308
Catalonia	7 675 217	16.32	4.5	965
Valencian Community	5 003 769	10.64	4.3	912
Extremadura	1 067 710	2.27	0.8	164
Galicia	2 699 499	5.74	3.6	761
Madrid, Community of	6 663 394	14.16	10.0	2116
Murcia, Region of	1 493 898	3.17	4.2	892
Navarre, Chartered Community of	654 214	1.39	0.7	150
Basque Country	2 207 776	4.69	4.7	985
Rioja, La	316 798	0.67	0.5	103
Ceuta	84 777	0.18	0.1	14
Melilla	86 487	0.18	0.2	41

We feel it is important to contextualize the atmosphere days before the survey, since it may have influenced the responses obtained. On 14 March, the government declared a state of alarm and issued a 14-day lockdown order, thus revealing to the population the seriousness of the COVID-19 pandemic. Later, on 22 March an extension of the lockdown for a further 14 days was announced, in a more intense effort to control the infection (**Figure 1**).

### Assessments

An ad hoc sociodemographic and clinical data online questionnaire with the following information: age, sex, province of residence, education, marital status, living arrangement, work status, monthly income, changes in work status due to COVID-19, changes in monthly income due to COVID-19, number and age of dependent children, and dependent older adults, questions about carrying out different activities during lockdown, current underlying conditions, such us hypertension or diabetes, and past / current mental disorders (type of disorder, pharmacological and psychological treatment). COVID-19 variables included coronavirus testing, coronavirus retesting, number of days with COVID-19 symptoms, COVID-19 symptoms, hospitalization due to COVID-19, number of family members infected with coronavirus and relationship to them, and number of household members infected with coronavirus.

The Spanish versions of the Depression, Anxiety and Stress Scale (DASS-21) [18], and the Impact of Event Scale (IES) [19] were used to measure the psychological impact of COVID-19. The DASS-21 is a self-rated scale developed to assess symptoms of depression (items 3, 5, 10, 13, 16, 17, 21), anxiety (2, 4, 7, 9, 15, 19, 20), and stress (1, 6, 8, 11, 12, 14, 18) over the past week. It provides scores for each of these three subscales (range 0-7). The IES is a 15-item self-rated scale that assesses current subjective distress related to a specific event. It has two subscales: intrusion (1, 4, 5, 6, 10, 11, 14) and avoidance (2, 3, 7, 8, 9, 12, 13, 15) and provides scores for each subscale (0-7 and 0-8, respectively) and a total score (0-15). Subjects were asked to think about whether they had experienced any of the psychological symptoms found in these questionnaires during the last week and in relation to the COVID-19 pandemic and lockdown.

In this study, we have simplified the scoring options of the two scales for the following two reasons. First, the validation process does not provide cut-off scores for the Spanish population. Furthermore, the representativeness of the sample employed in the process is at least doubtful (DASS-21 sample: 365 psychology students and 35 patients; IES sample: 1.078 students). Second, we wanted to avoid any self-report bias and make the survey friendlier for the general population, as most of them would complete the scales on their own devices. Thus, instead of the four response options (0-3), two options (0 “no”, 1 “yes”) were used. Higher scores on the five subscales mean greater distress. In addition, subscale scores from 0 to 3 were considered “not a case” while scores from 4 to 7 (or 8, avoidance scale) were considered “a probable case” of depressive, anxiety, stress, intrusive, or avoidance responses, respectively. DASS-21 subscale scores also reflect the severity of each of the three maladaptive responses. Thus, we considered scores of 0-1 “no maladaptive response”, 2-3 “doubtful”, 4 “mild”, 5 “moderate”, 6 “severe”, and 7 “extremely severe” maladaptive response.

The online questionnaire did not allow people to leave questions blank, thus we did not have to deal with missing data. However, the questionnaire does not provide us the number of people who started the survey but did not send it.

### Data analysis

Analyses were performed using IBM SPSS Statistics for Windows, Version 24.0 (IBM, Armonk, NY, USA). The significance level was set at *P* < 0.05.

Respondents were classified into 3 groups based on the yes/no answers to past and current mental health problems questions. Those were validated using the answers to a new question “What kind?”. We established the criterion that if a mismatch was identified between the two questions (current/past mental health problem and what type), the answer to the second question would prevail. Therefore, those who answered “No” to both questions were classified as “Never Mental Disorder group (NMD)”, those who answered “Yes” to the current mental health problem question, regardless of their answer to the question of past mental health problem, were classified as “Current Mental Disorder group (CMD)”, and those who answered “Yes” to past mental health problem and “No” to current as “group of Past Mental Disorder (PMD)”.

Means and standard deviations and frequencies and percentages were used to describe respondents' characteristics. We used a χ^2^ test and an ANOVA with Duncan post hoc analysis to identify differences between groups and to identify those variables associated with being/not being considered a case of any of the five psychological domains assessed. For each of the domains, we performed two binary logistic regressions. In the first regression, we obtained the predictive variables by introducing the relevant independent variables identified in the univariate analyses using a backward stepwise method. In the second, we introduced the predictive variables identified in the first model using the enter method. For each of the five models obtained, we show the model χ^2^ test, the Cox and Snell R^2^, the Nagelkerke R^2^, the Hosmer and Lemeshow χ-square test, and the percentage of correct predictions, and for each of the categories of the variables in the model, we show the B coefficient and its *P*-value, and the odds ratio (*OR*) with its 95% confidence interval (95% confidence interval, CI) (see Tables S1 to S5 in the **Online Supplementary Document**). To facilitate reading only the OR values and their 95% CI are shown in the tables.

## RESULTS

### Sociodemographic characteristics of the whole sample and by participant mental status

The sociodemographic and clinical characteristics of the sample as a whole and each of the three groups identified according to absence or presence of past / current mental disorder are shown in **Table 2**. The three groups differ significantly in all variables, except for three of them (**Table 2**). For this reason, we decided to analyse the psychological impact of the COVID-19 pandemic and lockdown in each of the three groups separately.

**Table 2:** Sociodemographic and clinical characteristics for the whole sample and according to the mental state of the participants

	Total sample N = 21207	Never Mental Disorder N = 15053	Past Mental Disorder N = 3665	Current Mental Disorder N = 2489	Statistical test, *P*
**Sociodemographic variables**					
**Age** (Mean, SD)	39.7 (14.0)	40.1 (14.3)	40.2 (13.3)	37.0 (13.3)	56.060*, <0.001
**Gender**, female (n, %)	14 768 (69.6)	9914 (65.9)	2830 (77.2)	2024 (81.3)	361.736†, <0.001
**Civil status (n, %):**					113.393†, <0.001
Never married	9867 (46.5)	6876 (45.7)	1654 (45.1)	1337 (53.7)	
Married/Living as married	9630 (45.4)	7071 (47.0)	1625 (44.3)	934 (37.5)	
Separated/Divorced/Widowed	1710 (8.1)	1106 (7.3)	386 (10.5)	218 (8.8)	
**Education level (n, %):**					170.051†, <0.001
Primary	333 (1.6)	214 (1.4)	53 (1.4)	66 (2.7)	
Secondary	7688 (36.3)	5272 (35.0)	1251 (34.1)	1165 (46.8)	
University	13 186 (62.2)	9567 (63.6)	2361 (64.4)	1258 (50.5)	
**Work status (n, %):**					313.554†, <0.001
Unemployed	1829 (8.6)	1134 (7.5)	376 (10.3)	319 (12.8)	
Working:					
-Employed	7679 (36.2)	5660 (37.6)	1284 (35.0)	735 (29.5)	
-Self-employed	2048 (9.7)	1480 (9.8)	358 (9.8)	210 (8.4)	
-Civil servant	4099 (19.3)	2988 (19.8)	756 (20.6)	355 (14.3)	
-Retired	1312 (6.2)	961 (6.4)	224 (6.1)	127 (5.1)	
-Student/Housewife	3392 (16.0)	2246 (14.9)	534 (14.6)	612 (24.6)	
Other	848 (4.0)	584 (3.9)	133 (3.6)	131 (5.3)	
**Income (€) (n, %):**					433.397†, <0.001
No income	3349 (15.8)	2229 (14.8)	515 (14.1)	605 (24.3)	
Less than 500	1462 (6.9)	930 (6.2)	268 (7.3)	264 (10.6)	
500-999	2667 (12.6)	1749 (11.6)	517 (14.1)	401 (16.1)	
1000-1499	4201 (19.8)	2947 (19.6)	789 (21.5)	465 (18.7)	
1500-1999	3799 (17.9)	2822 (18.7)	671 (18.3)	306 (12.3)	
More than 1999	4404 (20.8)	3422 (22.7)	698 (19.0)	284 (11.4)	
Refuse to answer	1325 (6.2)	954 (6.3)	207 (5.6)	164 (6.6)	
**Change in work status due to COVID-19 (n, %):**					29.454†, <0.001
No	17 764 (84.7)	12 704 (85.3)	3017 (83.3)	2043 (83.2)	
ETLA/EPLO‡	1871 (8.9)	1308 (8.8)	343 (9.5)	220 (9.0)	
Dismissal	390 (1.9)	238 (1.6)	82 (2.3)	70 (2.9)	
Forced vacation	954 (4.5)	649 (4.4)	182 (5.0)	123 (5.0)	
**Change in income due to COVID-19 (n, %):**					40.281†, <0.001
No	15 677 (73.9)	11 283 (75.0)	2607 (71.1)	1787 (71.8)	
Reduction, up to 25%	2292 (10.8)	1601 (10.6)	432 (11.8)	259 (10.4)	
Reduction, 26%-50%	1367 (6.4)	921 (6.1)	264 (7.2)	182 (7.3)	
Reduction, 51%-100%	1738 (8.2)	1151 (7.6)	342 (9.3)	245 (9.8)	
Increase	133 (0.6)	97 (0.6)	20 (0.5)	16 (0.6)	
**Living situation (n, %):**					43.823†, <0.001
Alone	2580 (12.2)	1716 (11.4)	544 (14.8)	320 (12.9)	
Two people	7534 (35.5)	5322 (35.4)	1330 (36.3)	882 (35.4)	
Three to five	10 722 (50.6)	7734 (51.4)	1739 (47.4)	1249 (50.2)	
More than five	371 (1.7)	281 (1.9)	52 (1.4)	38 (1.5)	
**Children in your charge (n, %):**					70.779†, <0.001
No	14 207 (67.0)	9942 (66.0)	2452 (66.9)	1813 (72.8)	
One	3357 (15.8)	2357 (15.7)	638 (17.4)	362 (14.5)	
Two	3050 (14.4)	2293 (15.2)	489 (13.3)	268 (10.8)	
More than two	593 (2.8)	461 (3.1)	86 (2.3)	46 (1.8)	
**Elderly in your charge (n, %):**					35.097†, <0.001
No	19203 (90.6)	13710 (91.1)	3313 (90.4)	2180 (87.6)	
One	1379 (6.5)	932 (6.2)	241 (6.6)	206 (8.3)	
Two	521 (2.5)	335 (2.2)	96 (2.6)	90 (3.6)	
More than two	104 (0.5)	76 (0.5)	15 (0.4)	13 (0.5)	
**Able to enjoy free time (n, %):**					590.492†, <0.001
No	1605 (7.6)	874 (5.8)	242 (6.6)	489 (19.7)	
Yes	19 571 (92.4)	14 156 (94.2)	3418 (93.4)	1997 (80.3)	
**March day that they responded to the survey (n, %):**					81.723†, <0.001
19	5763 (27.2)	4162 (27.6)	973 (26.5)	628 (25.2)	
20	3735 (17.6)	2704 (18.0)	634 (17.3)	397 (16.0)	
21	1640 (7.7)	1138 (7.6)	296 (8.1)	206 (8.3)	
22	1432 (6.8)	1000 (6.6)	249 (6.8)	183 (7.4)	
23	1804 (8.5)	1188 (7.9)	328 (8.9)	288 (11.6)	
24	635 (3.0)	413 (2.7)	109 (3.0)	113 (4.5)	
25	1203 (5.7)	833 (5.5)	221 (6.0)	149 (6.0)	
26	4995 (23.6)	3615 (24.0)	855 (23.3)	525 (21.1)	
**Somatic disease and COVID-19 variables**					
**Current somatic disease§ (n, %):**					255.319†, <0.001
No	14 017 (71.8)	10 435 (74.5)	2309 (69.2)	1273 (58.4)	
Yes	5514 (28.2)	3576 (25.5)	1030 (30.8)	908 (41.6)	
**Days with COVID-19 symptoms (n, %):**					65.601†, <0.001
None	18 761 (88.5)	13 476 (89.5)	3181 (86.8)	2104 (84.5)	
One-two days	1143 (5.4)	735 (4.9)	225 (6.1)	183 (7.4)	
Three to five	600 (2.8)	383 (2.5)	124 (3.4)	93 (3.7)	
Six to fourteen	559 (2.6)	368 (2.4)	107 (2.9)	84 (3.4)	
More than fourteen	144 (0.7)	91 (0.6)	28 (0.8)	25 (1.0)	
**Taken COVID-19 test (n, %):**					4.912†, 0.555
No	20 894 (98.6)	14 827 (98.5)	3609 (98.5)	2458 (98.8)	
Yes, negative result	180 (0.8)	133 (0.9)	29 (0.8)	18 (0.7)	
Yes, positive result	64 (0.3)	49 (0.3)	11 (0.3)	4 (0.2)	
Yes, waiting for result	59 (0.3)	37 (0.2)	14 (0.4)	8 (0.3)	
**Family/Friends infected by COVID-19 (n, %):**					6.647†, 0.355
No	16 669 (78.7)	11 863 (78.9)	2853 (78.0)	1953 (78.6)	
One	2181 (10.3)	1517 (10.1)	383 (10.5)	281 (11.3)	
Two	1184 (5.6)	842 (5.6)	213 (5.8)	129 (5.2)	
More than two	1137 (5.4)	804 (5.4)	211 (5.8)	122 (4.9)	
**Living with people infected by COVID-19 (n, %):**					10.102†, 0.120
No	20 848 (98.3)	14 790 (98.3)	3614 (98.6)	2444 (98.2)	
One	251 (1.2)	180 (1.2)	38 (1.0)	33 (1.3)	
Two	46 (0.2)	40 (0.3)	5 (0.1)	1 (0.0)	
More than two	62 (0.3)	43 (0.3)	8 (0.2)	11 (0.4)	

*ANOVA test (Duncan post-hoc: people with a current mental disorder are significantly younger than the other two groups, which do not differ from each other).

†χ^2^ test. SD: standard deviation.

‡ETLA: Employee Temporary Lay Off. EPLO: Employee Permanent Lay Off.

§Somatic disease includes: Hypertension, diabetes, cardiovascular diseases, respiratory diseases (asthma, chronic obstructive pulmonary disease, etc), and cancer.

### The psychological impact of the COVID-19 pandemic and the lockdown for the whole sample and by participant mental status

We found a statistically significant gradient in the psychological impact experienced according to the mental state of the respondent (NMD<PMD<CMD) (**Table 3**).

**Table 3:** Psychological impact of the COVID19 pandemic and the confinement for the whole sample and according to the mental state of the participants.

	Total simple, N = 21 207	Never Mental Disorder, N = 15 053 (71.0%)	Past Mental Disorder, N = 3665 (17.3%)	Current Mental Disorder, N = 2489 (11.7%)	Statistical test, *P*
**DASS-21 subscales (mean, SD)**					
Depression	3.6 (1.1)	3.5 (1.0)	3.8 (1.1)	4.3 (1.2)	696.442*, <0.001
Anxiety	1.2 (1.6)	0.9 (1.3)	1.3 (1.6)	2.8 (2.1)	1766.363*, <0.001
Stress	2.4 (2.4)	2.1 (2.2)	2.6 (2.4)	4.2 (2.4)	968.906*, <0.001
**DASS-21 subscales (n, %)**					
**Depression**					1569.842†, <0.001
No	442 (2.1)	362 (2.4)	52 (1.4)	28 (1.1)	
Doubtful	10 852 (51.2)	8532 (56.7)	1712 (46.7)	608 (24.4)	
Mild	5940 (28.0)	4031 (26.8)	1108 (30.2)	801 (32.2)	
Moderate	2655 (12.5)	1535 (10.2)	525 (14.3)	595 (23.9)	
Severe	1003 (4.7)	464 (3.1)	204 (5.6)	335 (13.5)	
Extremely severe	315 (1.5)	129 (0.9)	64 (1.7)	122 (4.9)	
**Depression**					1011.459†<0.001
No	11 294 (53.3)	8894 (59.1)	1764 (48.1)	636 (25.6)	
Yes	9913 (46.7)	6159 (40.9)	1901 (51.9)	1853 (74.4)	
**Anxiety**					2981.912†, <0.001
No	14 825 (69.9)	11 624 (77.2)	2362 (64.4)	839 (33.7)	
Doubtful	4111 (19.4)	2510 (16.7)	876 (23.9)	725 (29.1)	
Mild	970 (4.6)	456 (3.0)	212 (5.8)	302 (12.1)	
Moderate	672 (3.2)	276 (1.8)	118 (3.2)	278 (11.2)	
Severe	384 (1.8)	131 (0.9)	64 (1.7)	189 (7.6)	
Extremely severe	245 (1.2)	56 (0.4)	33 (0.9)	156 (6.3)	
**Anxiety**					2158.782†, <0.001
No	18 936 (89.3)	14 134 (93.9)	3238 (88.3)	1564 (62.8)	
Yes	2271 (10.7)	919 (6.1)	427 (11.7)	925 (37.2)	
**Stress**					1876.631†, <0.001
No	9842 (46.4)	7809 (51.9)	1551 (42.3)	482 (19.4)	
Doubtful	4314 (20.3)	3151 (20.9)	805 (22.0)	358 (14.4)	
Mild	1907 (9.0)	1267 (8.4)	351 (9.6)	289 (11.6)	
Moderate	1814 (8.6)	1106 (7.3)	352 (9.6)	356 (14.3)	
Severe	1827 (8.6)	994 (6.6)	334 (9.1)	499 (20.0)	
Extremely severe	1503 (7.1)	726 (4.8)	272 (7.4)	505 (20.3)	
**Stress**					1480.478†, <0.001
No	14 156 (66.8)	10 960 (72.8)	2356 (64.3)	840 (33.7)	
Yes	7051 (33.2)	4093 (27.2)	1309 (35.7)	1649 (66.3)	
**IES subscales (mean, SD)**					
Intrusion	2.12 (1.9)	1.9 (1.8)	2.3 (1.9)	3.3 (2.1)	668.420*, <0.001
Avoidance	3.29 (2.0)	3.0 (2.0)	3.5 (2.0)	4.4 (2.0)	570.820*, <0.001
Total IES	5.41 (3.4)	4.9 (3.2)	5.8 (3.3)	7.8 (3.5)	858.696*, <0.001
**IES subscales (n, %)**					
**Intrusion**					945.975†, <0.001
No	16 208 (76.4)	12 179 (80.9)	2713 (74.0)	1316 (52.9)	
Yes	4999 (23.6)	2874 (19.1)	952 (26.0)	1173 (47.1)	
**Avoidance**					723.619†, <0.001
No	11 806 (55.7)	9117 (60.6)	1885 (51.4)	804 (32.2)	
Yes	9401 (44.3)	5936 (39.4)	1780 (48.6)	1685 (67.7)	

SD – standard deviation, DASS-21- Depression, Anxiety and Stress Scale (No: includes No and Doubtful; Yes: includes Mild, Moderate, Severe, and Extremely severe), IES – Impact of Event Scale

*ANOVA test (Duncan post-hoc: on all subscales, the three groups differ significantly from each other).

†χ^2^ test.

On the DASS-21, in all three groups, depressive symptoms were the most prevalent (NMD = 40.9%, PMD = 51.9%, CMD = 74.4%, *F* = 1011.459, *P* < 0.001) and anxious symptoms the least prevalent (NMD = 6.1%, PMD = 11.7%, CMD = 37.24%, *F* = 2158.782, *P* < 0.001). On the IES, the most prevalent style was avoidance style (**Table 3**).

**Figure 2:**
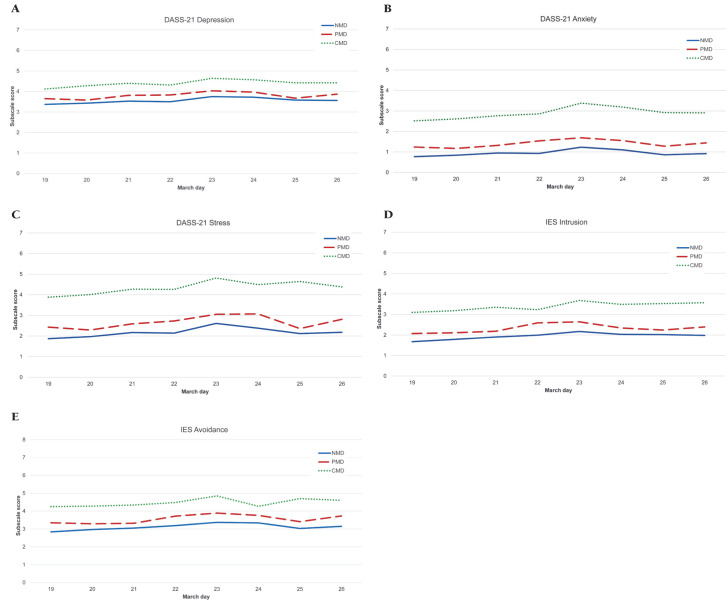
Psychological impact according to the number of days elapsed between the declaration of the state of alarm and the confinement in Spain (March 14) and the response to our questionnaire (March 19 – March 26) for the three mental state groups. **Panel A**. Depression, Anxiety and Stress subscale scores. Blue solid line: Never mental disorder (NMD); red dashed line: Past mental disorder (PMD); green dotted line: Current mental disorder (CMD). *Panel A Depression*. DASS-21: Depression, Anxiety and Stress Scale, NMD: Never mental disorder, PMD: Past mental disorder, CMD: Current mental NMD: *F =* 26.642, *P* < 0.001; PMD: *F =* 10.010, *P* < 0.001; CMD: *F =* 6.187, *P* < 0.001. *Panel A Anxiety*. DASS-21: Depression, Anxiety and Stress Scale, NMD: Never mental disorder, PMD: Past mental disorder, CMD: Current mental. NMD: *F =* 18.206, *P* < 0.001; PMD: *F =* 4.933, *P* < 0.001; CMD: *F =* 5.877, *P* < 0.001. *Panel A Stress*. DASS-21: Depression, Anxiety and Stress Scale, NMD: Never mental disorder, PMD: Past mental disorder, CMD: Current mental. NMD: *F =* 18.313, *P* < 0.001; PMD: *F =* 6.006, *P* < 0.001; CMD: *F =* 6.151, *P* < 0.001. **Panel B.** Impact of Event subscale scores. Blue solid line: Never mental disorder (NMD); red dashed line: Past mental disorder (PMD); green dotted line: Current mental disorder (CMD). *Panel B Intrusion*. IES: Impact of Event Scale, NMD: Never mental disorder, PMD: Past mental disorder, CMD: Current mental. NMD: *F =* 16.566, *P* < 0.001; PMD: *F =* 5.332, *P* < 0.001; CMD: *F =* 3.660, *P* = 0.001. *Panel B Avoidance*. IES: Impact of Event Scale, NMD: Never mental disorder, PMD: Past mental disorder, CMD: Current mental. NMD: *F =* 16.566, *P* < 0.001; PMD: *F =* 5.332, *P* < 0.001; CMD: *F =* 3.660, *P* = 0.001.

We also explored the evolution of psychological impact according to the number of days elapsed between the declaration of the state of alarm and the lockdown order in Spain (14 March) and the response to our questionnaire (19-26 March). In all three groups, psychological impact increased progressively and significantly until 23 March and then decreased slightly (**Figure 2**).

### Factors associated with the psychological impact of the COVID-19 pandemic and the lockdown by participant mental status

#### Never Mental Disorder group (NMD)

Most of the sociodemographic variables had a significant impact on psychological response (**Table 3** and Tables S1 to S5 in the **Online Supplementary Document**). Regarding non-modifiable factors, being female confers a greater risk of pathological responses in the five psychological domains, while advancing age decreases the risk of depression (OR = 0.981) or stress (OR = 0.981) and increases the risk of intrusive thoughts (OR = 1.006). Furthermore, being single or married has some protective effect against depression (OR = 0.771 and 0.784, respectively), but being married also confers a risk of stress (OR = 1.254). Regarding working status, being self-employed protects against depression (OR = 0.787), being retired against stress (OR = 0.583), and being a student or housewife against intrusive thoughts (OR = 0.729) (**Table 3**). An income≥€1,500/month (US$1685.26/month) is associated with a lower risk of depression (OR_€1500-1999 (US$1685.26-2245.89)_ = 0.872 > OR_€1999 (US$2245.89)_ = 0.765) and any reduction in income with a higher risk of depression and intrusive thoughts. Living with 1 to 4 more people protects against depression and intrusive thoughts, but living with two or more people is also associated with higher levels of stress. Having dependent children increases intrusive thoughts, but reduces the likelihood of an avoidance response (**Table 4**). However, having dependent older adults is not associated with any psychological impact. Finally, being able to enjoy free time protects against anxiety, stress, intrusive thoughts, and avoidance, but not against depression.

**Table 4:** Factors associated with the psychological impact of the COVID-19 pandemic and the confinement in the “Never mental disorder” group

	DASS-21	IES			
	**Depression**	**Anxiety**	**Stress**	**Intrusion**	**Avoidance**
	**OR (95% CI)**	**OR (95% CI)**	**OR (95% CI)**	**OR (95% CI)**	**OR (95% CI)**
**Sociodemographic variables**					
**Age**	0.981 (0.977-0.986)		0.971 (0.966-0.977)	1.006 (1.001-1.012)	
**Gender, reference: Male**					
Female	1.631 (1.500-1.773)	1.529 (1.239-1.865)	1.191 (1.077-1.316)	1.371 (1.224-1.535)	1.732 (1.598-1.878)
**Civil status, reference: Separated/ Divorced/Widowed**					
Never married	0.771 (0.649-0.916)				
Married/Living as married	0.784 (0.671-0.915)		1.254 (1.023-1.537)		
**Education, reference: University**					
Primary		2.295 (1.201-4.385)	0.871 (0.788-0.963)		1.660 (1.288-2.245)
Secondary	0.874 (0.810-0.955)				1.260 (1.159-1.369)
**Work status, reference: Unemployed**					
Self-employed	0.787 (0.635-0.975)				
Retired			0.583 (0.409-0.830)		
Student/Housewife				0.729 (0.579-0.917)	
**Income (€), reference: No income**					
500-999					0.854 (0.744-0.981)
1000-1499					0.820 (0.724-0.928)
1500-1999					0.769 (0.674-0.876)
More than 1999	0.765 (0. 618-0.945)				0.641 (0.560-0.734)
**Change in income due to COVID-19, reference: No**					
Reduction, up to 25%	1.189 (1.048-1.350)			1.283 (1.071-1.537)	
Reduction, 26%-50%	1.224 (1.036-1.446)				
Reduction, 51%-100%	1.234 (1.041-1.462)			1.381 (1.89-1.750)	
**Living situation, reference: Alone**					
Two people	0.742 (0.649-0.849)				
Three to five	0.760 (0.657-0.878)		1.380 (1.163-1.637)		
More than five			1.645 (1.163-2.327)	0.560 (0.363-0.865)	
**Children in your charge, reference: No**					
One				1.191 (1.022-1.387)	0.848 (0.764-0.941)
Two				1.280 (1.084-1.512)	0.755 (0.678-0.841)
More than two					0.694 (0.554-0.868)
**Able to enjoy free time, reference: No**					
Yes		0.490 (0.400-0.600)	0.275 (0.230-0.329)	0.488 (0.379-0.529)	0.655 (0.557-0.770)
**March day that they responded to the survey, reference: 19**					
20	1.127 (1.006-1.263)				1.132 (1.015-1263)
21	1.215 (1.043-1.415)				
22				1.306 (1.069-1.596)	1.198 (1.026-1.398)
23	1.479 (1.272-1.720)			1.300 (1.079-1.566)	
24	1.383 (1.097-1.742)	1.646 (1.079-2.510)			1.301 (1.039-1.629)
25	1.359 (1.144-1.615)			1.431 (1.160-1.765)	
26	1.350 (1.216-1.499)				1.119 (1.012-1.238)
**Somatic disease and COVID-19 variables**					
Current somatic disease*, reference: No					
Yes		1.337 (1.154-1.642)			
**Days with COVID-19 symptoms, reference: None**					
One-two days		1.843 (1.392-2.442)			
Three to five		2.001 (1.361-2.943)			
Six to fourteen		2.631 (1.818-3.808)			
More than fourteen					
**Family/Friends infected by COVID-19, reference: No**					
Yes	1.185 (1.081-1.299)		1.164 (1.049-1.293)	1.158 (1.033-1.298)	
**Psychological variables**					
**DASS-21 Depression case, reference: No**					
Yes		1.789 (1.475-2.170)	3.429 (3.129-3.757)	2.266 (2.041-2.515)	1.801 (1.666-1.946)
**DASS-21 Anxiety case, reference: No**					
Yes	1.504 (1.250-1.808)		7.286 (5.907-8.988)	3.286 (2.785-3.877)	1.495 (1.262-1.771)
**DASS-21 Stress case, reference: No**					
Yes	3.395 (3.100-3.719)	7.266 (5.852-9.023)		3.626 (3.264-4.028)	1.969 (1.800-2.154)
**IES Intrusion case, reference: No**					
Yes	2.211 (1.993-2.453)	3.475 (2.931-4.120)	3.611 (3.254-4.007)		2.364 (2.141-2.161)
**IES Avoidance case, reference: No**					
Yes	1.792 (1.657-1.938)	1.584 (1.325-1.893)	1.961 (1.790-2.147)	2.468 (2.233-2.728)	

OR – odds ratio, CI – confidence interval, DASS-21 – Depression, Anxiety and Stress Scale (No: includes No and Doubtful; Yes: includes Mild, Moderate, Severe, and Extremely severe), IES – Impact of Event Scale

*Somatic disease includes: Hypertension, diabetes, cardiovascular diseases, respiratory diseases (asthma, chronic obstructive pulmonary disease, etc), and cancer.

Regarding the variables related to physical health and COVID-19, having an underlying condition (OR = 1.337) and COVID-19 symptoms for 1 to 14 days (OR_1-2 days_ = 1.843; OR_3-5 days_ = 2.001; 6-14 days = 2.631) increases the risk of anxiety, while having family members or friends infected with coronavirus is associated with a depressive coping style (OR = 1.185) and intrusive thoughts (OR = 1.158) (see **Table 4** and Tables S1, S2, and S4 of the **Online Supplementary Document**).

#### Past Mental Disorder group (PMD)

As in the NMD group, being female in this population increases the risk of having a depressive coping style (OR = 1.419), intrusive thoughts (OR = 1.281), and avoidance (OR = 1.754). Also, advancing age decreases the risk of depression (OR = 0.990), stress (OR = 0.962), and avoidance (OR = 0.992) but increases the risk of intrusive thoughts (OR = 1.011). While being single is associated with a lower risk of anxiety (OR = 0.653), being married does so with a higher risk of stress (OR = 1.933). Also, being retired protects against depression (OR = 0.641), but being employed or being a civil servant is associated with stress (OR = 1.450 and 1.482, respectively) (see **Table 5** and Tables S1-S5 of the **Online Supplementary Document**). In this group, income, change in income due to COVID-19, living situation, and having dependent children or older adults do not have an impact on psychological state. Ability to enjoy free time reduces the risk of anxiety (OR = 0.685), stress (OR = 0.215), and intrusive thoughts (OR = 0.496).

**Table 5:** Factors associated with the psychological impact of the COVID pandemic and the confinement in the “Past mental disorder” group

	DASS-21	IES			
	**Depression**	**Anxiety**	**Stress**	**Intrusion**	**Avoidance**
	**OR (95% CI)**	**OR (95% CI)**	**OR (95% CI)**	**OR (95% CI)**	**OR (95% CI)**
**Sociodemographic variables**					
**Age**	0.990 (0.983-0.997)		0.962 (0.953-0.972)	1.011 (1.003-1.020)	0.992 (0.986-0.998)
**Gender, reference: Male**					
Female	1.419 (1.190-1.691)			1.281 (1.029-1.595)	1.754 (1.471-2.091)
**Civil status, reference: Separated/ Divorced/Widowed**					
Never married		0.653 (0.511-0.834)			
Married/Living as married			1.933 (1.381-2.705)	1.524 (1.104-2.105)	
**Education, reference: University**					
Primary					
Secondary					1.466 (1.257-1.709)
**Work status, reference: Unemployed**					
Employed			1.450 (1.080-1.947)		
Civil servant			1.482 (1.073-2.047)		
Retired	0.641 (0.422-0.975)				
**Able to enjoy free time, reference: No**					
Yes		0.685 (0.490-0.958)	0.215 (0.149-0.313)	0.496 (0.366-0.672)	
**March day that they responded to the survey, reference: 19**					
20					
21					
22	1.422(1.066-1.897)			1.543 (1.088-2.188)	1.408 (1.033-1.919)
23				1.797 (1.305-2.474)	
24	1.707 (1.272-2.289)				
25					
26	1.248 (1.014-1.535)				1.346 (1.099-1.649)
**Somatic disease and COVID-19 variables**					
**Days with COVID-19 symptoms, reference: None**					
One-two days					
Three to five		2.546 (1.524-4.256)			
Six to fourteen		3.441 (1.963-6.032)			
More than fourteen					
**Family/Friends infected by COVID-19, reference: No**					
Yes	1.267 (1.060-1.515)				
**Living with people infected by COVID-19, reference: No**					
Yes		2.411 (1.144-5.080)			
**Psychological variables**					
**DASS-21 Depression case, reference: No**					
Yes		2.342 (1.735-3.163)	2.890 (2.429-3.440)	2.088 (1.728-2.524)	1.462 (1.256-1.703)
**DASS-21 Anxiety case, reference: No**					
Yes	2.235 (1.658-3.013)		5.612 (4.153-7.583)	2.517 (1.975-3.208)	1.911 (1.456-2.509)
**DASS-21 Stress case, reference: No**					
Yes	2.906 (2.447-3.453)	5.939 (4.405-8.008)		3.170 (2.621-3.832)	2.048 (1.725-2.431)
**IES Intrusion case, reference: No**					
Yes	2.052 (1.698-2.479)	2.590 (2.024-3.314)	3.024 (2.502-3.656)		2.374 (1.978-2.848)
**IES Avoidance case, reference: No**					
Yes	1.462 (1.254-1.703)	2.007 (1.524-2.642)	2.037 (1.716-2.417)	2.433 (2.028-2.919)	

OR – odds ratio, CI – confidence interval, DASS-21 – Depression, Anxiety and Stress Scale (No: includes No and Doubtful; Yes: includes Mild, Moderate, Severe, and Extremely severe), IES – Impact of Event Scale

Having experienced the symptoms of COVID-19 for 5 to 14 days and living with someone infected with COVID-19 increases the risk of anxiety (OR_3-5 days_ = 2.546; OR_6-14 days_ = 3.441; living with someone infected with coronavirus = 2.411). On the other hand, having family or friends infected with coronavirus is associated with a depressive response (OR = 1.267).

#### Current Mental Disorder group (CMD)

As in the others, being female is associated with an increased risk of stress (OR = 1.333) and having an avoidant response (OR = 1.879). Furthermore, advancing age decreases the risk of pathological responses in all domains, except for intrusive thoughts (see **Table 6** and Tables S1-S5 of the **Online Supplementary Document**). Being married is associated with a lower risk of depression (OR = 0.625), as is working as a civil servant (OR = 0.578). However, being employed increases the risk of stress (OR = 1.440) while being a student or housewife protects against intrusive thoughts (OR = 0.664). Living with 1 to 4 other people and having dependent children increases the risk of stress and intrusive thoughts. However, having two dependent children reduces the risk of avoidance (OR = 0.694). In this group only, having dependent older adults impacts the psychological state; thus, having 1 dependent older adult increases the risk of stress (OR = 2.599). Once again, ability to enjoy free time is a protective factor against anxiety (OR = 0.464), stress (OR = 0.274), and intrusive thoughts (OR = 0.582).

**Table 6:** Factors associated with the psychological impact of the COVID pandemic and the confinement in the “Current mental disorder” group

	DASS-21	IES			
	**Depression**	**Anxiety**	**Stress**	**Intrusion**	**Avoidance**
	**OR (95% CI)**	**OR (95% CI)**	**OR (95% CI)**	**OR (95% CI)**	**OR (95% CI)**
**Sociodemographic variables**					
**Age**	0.981 (0.971-0.992)	0.979 (0.971-0.987)	0.983 (0.972-0.994)		0.986 (0.978-0.994)
**Gender, reference: Male**					
Female			1.333 (1.020-1.744)		1.879 (1.494-2.363)
**Civil status, reference: Separated/ Divorced/Widowed**					
Never married					
Married/Living as married	0.625 (0.435-0.897)				
**Education, reference: University**					
Primary					
Secondary					1.326 (1.094-1.606)
**Work status, reference: Unemployed**					
Employed			1.440 (1.003-2.065)		
Civil servant	0.578 (0.395-0.847)				
Student/Housewife				0.664 (0.460-0.960)	
**Living situation, reference: Alone**					
Two people			1.528 (1.084-2.155)		
Three to five			1.998 (1.426-2.798)		
More than five					
**Children in your charge, reference: No**					
One				1.680 (1.239-2.279)	
Two					0.694 (0.496-0.971)
More than two				2.290 (1.047-5.009)	
**Elderly in your charge, reference: No**					
One					
Two			2.599 (1.344-5.027)		
More than two					
**Able to enjoy free time, reference: No**					
Yes		0.464 (0.365-0.588)	0.274 (0.188-0.399)	0.582 (0.447-0.759)	
March day that responded					
to the survey, reference: 19					
20					
21					
22					
23	1.528 (1.30-2.267)	1.559 (1.101-2.209)			
24		2.072 (1.242-3.455)			
25			2.476 (1.488-4.118)		
26					
**Somatic disease and COVID-19 variables**					
Current somatic disease*, reference: No					
Yes				1.240 (1007-1.528)	
**Days with COVID-19 symptoms, reference: None**					
One-two days					
Three to five		1.943 (1.161-3.252)			
Six to fourteen					
More than fourteen		5.398 (1.810-16.099)			
**Psychological variables**					
**Past mental disorder history, reference: No**					
Yes	1.352 (1.026-1.781)	1.502 (1.122-2.012)			
**DASS-21 Depression case, reference: No**					
Yes		1.423 (1.086-1.864)	2.611 (2.052-3.322)	1.531 (1.185-1.977)	1.522 (1.230-1.885)
**DASS-21 Anxiety case, reference: No**					
Yes			6.821 (4.994-9.317)	3.287 (2.626-4.114)	1.884 (1.452-2.342)
**DASS-21 Stress case, reference: No**					
Yes	2.659 (2.101-3.365)	6.976 (5.117-9.511)		3.492 (2.708-4.504)	1.623 (1.305-2.019)
**IES Intrusion case, reference: No**					
Yes	1.625 (1.279-2.063)	3.340 (2.701-4.131)	3.654 (2.870-4.652)		2.411 (1.942-2.992)
**IES Avoidance case, reference: No**					
Yes	1.587 (1.284-1.962)	1.850 (1.451-2.358)	1.557 (1.245-1.948)	2.325 (1.842-2.935)	

OR – odds ratio, CI – confidence interval, DASS-21 – Depression, Anxiety and Stress Scale (No: includes No and Doubtful; Yes: includes Mild, Moderate, Severe, and Extremely severe), IES – Impact of Event Scale

*Somatic disease includes: Hypertension, diabetes, cardiovascular diseases, respiratory diseases (asthma, chronic obstructive pulmonary disease, etc), and cancer.

Compared with the other two groups, in this population, the variables related to physical health and COVID-19 issues have a minor psychological impact. Just having an underlying condition and having experienced COVID-19 symptoms for more than 2 days is associated with an increased risk of intrusive thoughts (OR = 1.240) and anxiety (for OR, see **Table 5**).

Finally, in the three groups, being identified as a probable case of pathological response in any of the five psychological domains confers a significantly higher risk of being considered a probable case in the other four domains: NMD: OR ranging from 1.495 to 7.286 (**Table 4**); PMD: OR = 1.462-5.939 (**Table 5**); CMD: OR = 1.305-6.976 (**Table 6**).

## DISCUSSION

To the best of our knowledge, this is the first study in Spain to explore the psychological impact of the COVID-19 pandemic and in the scientific literature to examine responses according to mental health status. First, we found that the percentage of respondents showing maladaptive responses in any of the five psychological domains studied was different depending on the absence or presence of past / current mental disorder. We identified a gradual psychological impact according to mental health status, confirming that along with having a current mental disorder, having had a past mental disorder is a risk factor for developing maladaptive responses when coping with outbreaks. Second, in our study, the depressive response was the most frequent among the three groups (41%- 74%), followed by avoidant behaviour (39%-68%) and stress (27%-66%). However, unexpectedly, anxiety was the least common (6%- 37.2%).

A significant increase in the psychological distress experienced was observed after the announcement of the extension of a further 14 days of lockdown. This data agrees with previous studies suggesting that a longer lockdown is associated with poorer mental health outcomes [20,21]; therefore, it would be advisable not to establish a specific time limit, such was the case in Wuhan, China.

The frequency of depressive symptoms was higher than reported for China [8] but lower than in one study during the SARS epidemic in Hong Kong [22]. Socioeconomic characteristics, such as less income, and financial loss due to the pandemic, were identified as risk factors for developing depressive symptoms in the group with no prior mental health problems. This is in line with previous studies showing that lower incomes and financial loss as a result of lockdown increase the risk of depressive symptoms [23] and distress [23-25]. One explanation could be that people with lower incomes are more likely to be affected by the temporary loss of income than those with higher incomes [20]. However, this relationship was not found in the other two groups, and there are no studies to compare these results. In that sense, people with past or current mental disorders could be more used to having financial problems or feel more protected due to government aid for at-risk populations.

The anxiety response was the least frequent psychological response in our sample, with much lower rates than those reported in China [8], although both studies used the DASS-21. The DASS-21 anxiety subscale focuses more on physical symptoms of anxiety, such as shortness of breath or rapid heart rate which may be similar to the symptoms of COVID-19. Therefore, it could be that, as opposed to the Chinese population, our Spanish sample reacted less physically to the pandemic and the lockdown or they attributed these symptoms to COVID-19 instead of anxiety. Another factor that may have influenced the differences is the threshold we established for being considered a case of anxious response, since there were no set cut-off points for Spain. If, instead of adopting a conservative stance favouring specificity over sensitivity, we had required the presence of 2 symptoms only for a case of anxiety, the percentage would have been 30.1% instead of 10.7%, a figure more similar to that reported in China [8]. However, the same would apply for the differences in the depressive percentages. In that case, if we lowered the threshold, the differences between the Spanish and Chinese figures would be even higher (97.1% vs 16.5%). In any case, and leaving aside the cut-off points used, it seems that the psychological response of both countries are different (Spain: depressive style; China: anxious type). We think that culture and lifestyle differences are most likely responsible for these differences. Spain is a Mediterranean country, with an outgoing population that conducts social and family life activities mainly in the streets. However, the Chinese community is more reserved and with life more centred inside the home.

Concerning stress response, family responsibilities increased the risk of developing stress symptoms regardless of mental health status; furthermore, working only increased stress symptoms in those with past or current mental health problems. These results support the fact that people with mental health problems experience work difficulties that need to be addressed regardless of their specific diagnosis and severity [24].

Non-modifiable factors play different roles in the psychological response. While younger age was found to be a protective factor for all psychological domains except for avoidant behaviours, being female was a risk factor for all psychological areas in the NMD group and for some in the other two groups. The reason for this lack of influence in the PMD and CMD groups may be related to the fact that in these two groups, as expected, the rate of females was significantly higher than in the NMD group (77.2% and 81.3%, respectively, vs 65.9%). Previous studies [8,24] have shown that being female is associated with an increased likelihood of developing depressive responses and stress symptoms. Furthermore, young people could be more affected because they are worried about their education [8] and future employment, together with separation from their friends [26,27].

Being able to enjoy free time did prove to be a protective factor against the development of any symptom, but surprisingly, not against a depressive response (except in CMD). Lockdown frequently involves boredom, frustration, and distress due to disruption of usual daily routines and reduced social contacts [20]. In that sense, keeping up with regular daily activities, seems to have a protective psychological effect. For that reason, people should be advised about what they should do, and practical and meaningful coping strategies should be provided.

We found that except for “days with COVID-19 symptoms” in the case of anxiety, the variables related to COVID-19 and physical health had almost no significant impact on psychological response in any of the three groups. Their low prevalence (equally in the three groups) could influence our results. As during the initial phase of the COVID-19 outbreak in China [8], COVID-19 symptoms seems to be a risk factor for developing anxiety.

With regard to mental health problems, in line with previous studies, having had a past mental disorder conferred a risk of developing depressive and anxious responses, as well as intrusive thoughts in people with a current mental disorder [9].

The results should be interpreted in the context of some limitations. First, although we employed the respondent-driven sampling to partially control the recruitment bias, the external validity of our sample in terms of representativeness and selection bias is the main limitation. Furthermore, our conclusions cannot be extrapolated to the Spanish general population, as Catalonia and Valencia were clearly underrepresented, while the Principality of Asturias and Cantabria were overrepresented. However, it is necessary to emphasize the large sample size and its multicentre characteristics. Balanzá et al. (2020) [28] have stated the need to adopt online surveys and remote data collection in order to understand and address consequences after the COVID-19. Second, although the people who completed the survey reported experiencing symptoms, it should not be forgotten that these diagnoses are psychometric. To confirm the diagnoses, a structured diagnostic interviews would be required. However, due to the current situation, this was not possible.

## CONCLUSIONS

The Spanish sample population predominantly responded with a depressive style. Sociodemographic factors had the greatest impact on those who had never experienced or who currently had a mental disorder. Being able to enjoy free time was identified as a protective factor against most pathological psychological responses. The COVID-19 variables had no psychological impact, except for presence of COVD-19 symptoms, which was identified as a risk factor for development of anxiety. Finally, being identified as a case in any of the psychological domains studied was a risk factor for being a case in the rest of the domains. This can help develop coping strategies that address the modifiable risk and protective factors for early implementation in future outbreaks.

## Additional material

Online Supplementary Document
