# Supplementary material for: Early psychological impact of the 2019 coronavirus disease (COVID-19) pandemic and lockdown in a large Spanish sample
Source: J Glob Health. 2020 Oct 8;10(2):020505. doi: 10.7189/jogh.10.020505 (PMC7567432; doi:10.7189/jogh.10.020505)
Supplement: Online Supplementary Document [file jogh-10-020505-s001.pdf]

Table S1. Depressive response according to the DASS Depression subscale: Variables retained in the Logistic Regression model in each of the three groups studied.

	**Never mental disorder N = 15026**		**Past mental disorder N = 3660**		**Current mental disorder N = 2486**	
	B, p	O.R. (95% CI)	B, p	O.R. (95% CI)	B, p	O.R. (95% CI)
**Sociodemographic variables**						
Age	-0.019, <0.001	0.981 (0.977-0.986)	-0.010, 0.006	0.990 (0.983-0.997)	-0.19, 0.001	0.981 (0.971-0.992)
Gender						
Male	Reference	Reference	Reference	Reference		
Female	-0.489, <0.001	1.631 (1.500-1.773)	0.350, <0.001	1.419 (1.190-1.691)		
Civil status						
Never married	-0.260, 0.003	0.771 (0.649-0.916)			-0.270, 0.184	0.763 (0.512-1.137)
Married/Living as married	-0.244, 0.002	0.784 (0.671-0.915)			-0.470, 0.011	0.625 (0.435-0.897)
Separated/Divorced/Widowed	Reference	Reference			Reference	Reference
Education level						
Primary	-0.160, 0.351	0.853 (0.610-1.192)				
Secondary	-0.134, 0.003	0.874 (0.810-0.955)				
University	Reference	Reference				
Work status						
Unemployed	Reference	Reference	Reference	Reference	Reference	Reference
Working						
Employed	-0.115, 0.199	0.891 (0.747-1.063)	-0.208, 0.114	0.812 (0.628-1.051)	-0.306, 0.082	0.736 (0.522-1.040)
Self-employed	-0.240, 0.029	0.787 (0.635-0.975)	-0.284, 0.087	0.753 (0.544-1.042)	-0.259, 0.248	0.772 (0.498-1.197)
Civil servant	-0.146, 0.142	0.864 (0.711-1.050)	-0.211, 0.141	0.810 (0.611-1.072)	-0.548, 0.005	0.578 (0.395-0.847)
Retired	-0.082, 0.535	0.921 (0.711-1.194)	-0.445, 0.038	0.641 (0.422-0.975)	-0.249, 0.357	0.780 (0.459-1.324)

Student/Housewife	0.057, 0.533	1.059 (0.885-1.267)	-0.219, 0.169	1.245 (0.911-1.701)	-0.058, 0.768	0.943 (0.640-1.391)
Other	-0.272, 0.024	0.762 (0.601-0.965)	0.057, 0.804	1.059 (0.675-1.660)	-0.014, 0.959	0.986 (0.578-1.683)
Income (€)						
No income	Reference	Reference				
Less than 500	0.036, 0.710	1.037 (0.858-1.253)				
500-999	0.017, 0.870	1.017 (0.834-1.239)				
1000-1499	-0.085, 0.403	0.918 (0.753-1.121)				
1500-1999	-0.136, 0.197	0.872 (0.709-1.073)				
More or equal to 2000	-0.268, 0.013	0.765 (0.618-0.945)				
Refuse to answer	-0.066, 0.542	0.936 (0.757-1.157)				
Change in income due to COVID-19						
No	Reference	Reference				
Reduction, up to 25%	0.173, 0.007	1.189 (1.048-1.350)				
Reduction, 26-50%	0.202, 0.018	1.224 (1.036-1.446)				
Reduction, 51-100%	0.210, 0.015	1.234 (1.041-1.462)				
Increase	-0.155, 0.515	0.857 (0.538-1.365)				
Living situation						
Alone	Reference	Reference				
Two people	-0.299, <0.001	0.742 (0.649-0.849)				
Three to five	-0.275, <0.001	0.760 (0.657-0.878)				
More than five	-0.283, 0.075	0.754 (0.553-1.029)				
Children in your charge						
No	Reference	Reference				
One	-0.076, 0.253	0.927 (0.813-1.056)				
Two	-0.141, 0.055	0.869 (0.752-1.003)				
More than two	-0.186, 0.146	0.830 (0.647-1.067)				
Able to enjoy free time						
No					Reference	Reference

Yes					0.257, 0.076	1.293 (0.973-1.718)
March day that responded to the survey						
19	Reference	Reference	Reference	Reference	Reference	Reference
20	0.120, 0.040	1.127 (1.006-1.263)	-0.133, 0.242	0.875 (0.700-1.094)	-0.080, 0.597	0.923 (0.685-1.243)
21	0.195, 0.012	1.215 (1.043-1.415)	0.352, 0.017	1.422 (1.066-1.897)	0.282, 0.160	1.326 (0.895-1.966)
22	0.045, 0.583	1.046 (0.890-1.230)	0.072, 0.654	1.075 (0.784-1.474)	-0.048, 0.812	0.953 (0.640-1.420)
23	0.392, <0.001	1.479 (1.272-1.720)	0.534, <0.001	1.707 (1.272-2.289)	0.424, 0.035	1.528 (1.030-2.267)
24	0.324, 0.006	1.383 (1.097-1.742)	0.201, 0.378	1.222 (0.782-1.909)	0.105, 0.690	1.111 (0.661-1.867)
25	0.307, <0.001	1.359 (1.144-1.615)	-0.001, 0.995	0.999 (0.722-1.383)	-0.193, 0.385	0.825 (0.533-1.275)
26	0.300, <0.001	1.350 (1.216-1.499)	0.221, 0.036	1.248 (1.014-1.535)	0.274, 0.066	1.315 (0.982-1.762)
**Somatic disease and COVID-19 variables**						
Days with COVID-19 symptoms						
None	-0.251, 0.300	0.778 (0.484-1.251)				
One-two days	-0.144, 0.575	0.866 (0.525-1.430)				
Three to five	-0.062, 0.818	0.940 (0.555-1.591)				
Six to fourteen	0.095, 0.724	1.100 (0.648-1.865)				
More than fourteen	Reference	Reference				
Family/Friends infected by COVID-19						
No	Reference	Reference	Reference	Reference		
Yes	0.170, <0.001	1.185 (1.081-1.299)	0.237, 0.009	1.267 (1.060-1.515)		
**Psychological variables**						
Past mental disorder						
No					Reference	Reference
Yes					0.301, 0.032	1.352 (1.026-1.781)
DASS-21 Anxiety						

No	Reference	Reference	Reference	Reference	Reference	Reference
Yes	0.408, <0.001	1.504 (1.250-1.808)	0.804, <0.001	2.235 (1.658-3.013)	0.266, 0.050	1.305 (1.000-1.704)
DASS-21 Stress						
No	Reference	Reference	Reference	Reference	Reference	Reference
Yes	1.222, <0.001	3.395 (3.100-3.719)	1.067, <0.001	2.906 (2.447-3.453)	0.978, <0.001	2.659 (2.101-3.365)
IES Intrusion						
No	Reference	Reference	Reference	Reference	Reference	Reference
Yes	0.793, <0.001	2.211 (1.993-2.453)	0.719, <0.001	2.052 (1.698-2.479)	0.485, <0.001	1.625 (1.279-2.063)
IES Avoidance						
No	Reference	Reference	Reference	Reference	Reference	Reference
Yes	0.583, <0.001	1.792 (1.657-1.938)	0.380, <0.001	1.462 (1.254-1.703)	0.462, <0.001	1587 (1.284-1.962)
Model Chi-Square [df], p	3678.172 [44], <0.001		788.821 [20], <0.001		391.290 [22], <0.001	
Cox and Snell's R^2	0.217		0.194		0.146	
Nagelkerke's R^2	0.293		0.259		0.214	
H&L[1] Chi-Square [df], p	66.257 [8], <0.001		10.523 [8], 0.230		8.245 [8], 0.410	
Correct predictions	72.6%		69.0%		77.3%	

1. Hosmer and Lemeshow test.

O.R.: Odds ratio. CI: Confidence interval. df: degrees of freedom

DASS-21: Depression, Anxiety and Stress Scale (No: includes No and Doubtful; Yes: includes Mild, Moderate, Severe, and Extremely severe). IES: Impact of Event Scale.

Table S2. Anxiety response according to the DASS Anxiety subscale: Variables retained in the Logistic Regression model in each of the three groups studied.

	**Never Mental Disorder group (NMD) N = 13989**		**Past Mental Disorder group (PMD) N = 3660**		**Current Mental Disorder group (CMD) N = 2486**	
	B, p	O.R. (95% CI)	B, p	O.R. (95% CI)	B, p	O.R. (95% CI)
**Sociodemographic variables**						
Age	-0.008, 0.036	0.993 (0.986-1.000)			-0.021, <0.001	0.979 (0.971-0.987)
Gender						
Male	Reference	Reference				
Female	0.419, <0.001	1.520 (1.239-1.865)				
Civil status						
Never married			-0.426, 0.001	0.653 (0.511-0.834)		
Married/Living as married			-0.360, 0.151	0.697 (0.426-1.141)		
Separated/Divorced/Widowed			Reference	Reference		
Education level						
Primary	0.831, 0.012	2.295 (1.201-4.385)	-0.662, 0.230	0.516 (0.175-1.522)		
Secondary	0.151, 0.085	1.163 (0.985-1.375)	0.236, 0.053	1.266 (0.997-1.609)		
University	Reference	Reference	Reference	Reference		
Able to enjoy free time						
No	Reference	Reference	Reference	Reference	Reference	Reference
Yes	-0.713, <0.001	0.490 (0.400-0.600)	-0.378, 0.027	0.685 (0.490-0.958)	-0.769, <0.001	0.464 (0.365-0.588)
March day that responded to the survey						
19	Reference	Reference			Reference	Reference
20	0.244, 0.057	1.276 (0.993-1.640)			0.055, 0.739	1.057 (0.762-1.466)
21	0.230, 0.148	1.259 (0.922-1.719)			0.275, 0.189	1.317 (0.873-1.986)
22	-0.197, 0.279	0.821 (0.575-1.173)			0.264, 0.212	1.302 (0.860-1.971)

23	0.167, 0.261	1.182 (0.883-1.582)			0.444, 0.012	1.559 (1.101-2.209)
24	0.498, 0.021	1.646 (1.079-2.510)			0.728, 0.005	2.072 (1.242-3.455)
25	-0.379, 0.070	0.685 (0.455-1.031)			0.015, 0.947	1.015 (0.651-1.583)
26	0.091, 0.435	1.095 (0.872-1.374)			0.096, 0.526	1.101 (0.817-1.483)
**Somatic disease and COVID-19 variables**						
Current somatic disease*						
No	Reference	Reference				
Yes	0.320, <0.001	1.337 (1.154-1.642)				
Days with COVID-19 symptoms						
None	Reference	Reference	Reference	Reference	Reference	Reference
One-two days	0.612, <0.001	1.843 (1.392-2.442)	0.349, 0.101	1.418 (0.934-2.152)	0.286, 0.127	1.331 (0.922-1.921)
Three to five	0.694, <0.001	2.001 (1.361-2.943)	0.935, <0.001	2.546 (1.524-4.256)	0.664, 0.011	1.943 (1.161-3.252)
Six to fourteen	0.968, <0.001	2.631 (1.818-3.808)	1.236, <0.001	3.441 (1.963-6.032)	0.472, 0.081	1.602 (0.943-2.722)
More than fourteen	0.671, 0.097	1.956 (0.885-4.326)	-0.152, 0.809	0.859 (0.250-2.953)	1.686, 0.002	5.398 (1.810-16.099)
Living with people infected by COVID-19						
No			Reference	Reference		
Yes			0.880, 0.021	2.411 (1.144-5.080)		
**Psychological variables**						
Past mental disorder						
No					Reference	Reference
Yes					0.407, 0.006	1.502 (1.122-2.012)
DASS-21 Depression						
No	Reference	Reference	Reference	Reference	Reference	Reference
Yes	0.582, <0.001	1.789 (1.475-2.170)	0.851, <0.001	2.342 (1.735-3.163)	0.353, <0.001	1.423 (1.086-1.864)
DASS-21 Stress						
No	Reference	Reference	Reference	Reference	Reference	Reference

Yes	1.983, <0.001	7.266 (5.852-9.023)	1.782, <0.001	5.939 (4.405-8.008)	1.942, <0.001	6.976 (5.117-9.511)
IES Intrusion						
No	Reference	Reference	Reference	Reference	Reference	Reference
Yes	1.246, <0.001	3.475 (2.931-4.120)	0.951, <0.001	2.590 (2.024-3.314)	1.206, <0.001	3.340 (2.701-4.131)
IES Avoidance						
No	Reference	Reference	Reference	Reference	Reference	Reference
Yes	0.460, <0.001	1.584 (1.325-1.893)	0.697, <0.001	2.007 (1.524-2.642)	0.615, <0.001	1.850 (1.451-2.358)
Model Chi-Square [df], p	1942.008 [21], <0.001		720.465 [14], <0.001		928.630 [18], <0.001	
Cox and Snell's R^2	0.130		0.179		0.312	
Nagelkerke's R^2	0.354		0.348		0.425	
H&L[1] Chi-Square [df], p	19.366 [8], 0.013		12.902 [8], 0.115		11.768 [8], 0.162	
Correct predictions	94.1%		88.9%		76.1%	

1. Hosmer and Lemeshow test.

O.R.: Odds ratio. CI: Confidence interval. df: degrees of freedom

* Somatic disease includes: Hypertension, diabetes, cardiovascular diseases, respiratory diseases (asthma, chronic obstructive pulmonary disease, etc), and cancer.

DASS-21: Depression, Anxiety and Stress Scale (No: includes No and Doubtful; Yes: includes Mild, Moderate, Severe, and Extremely severe). IES: Impact of Event Scale.

Table S3. Stress response according to the DASS Stress subscale: Variables retained in the Logistic Regression model in each of the three groups studied.

	Never Mental Disorder group (NMD) N = 15005		Past Mental Disorder group (PMD) N = 3660		Current Mental Disorder group (CMD) N = 2486	
	B, p	O.R. (95% CI)	B, p	O.R. (95% CI)	B, p	O.R. (95% CI)
**Sociodemographic variables**						
Age	-0.029, <0.001	0.971 (0.966-0.977)	-0.038, <0.001	0.962 (0.953-0.972)	-0.017, 0.002	0.983 (0.972-0.994)
Gender						
Male	Reference	Reference			Reference	Reference
Female	0.175, 0.001	1.191 (1.077-1.316)			0.288, 0.036	1.333 (1.020-1.744)
Civil status						
Never married	-0.114, 0.303	0.892 (0.718-1.109)	0.256, 0.173	1.292 (0.894-1.868)		
Married/Living as married	0.226, 0.030	1.254 (1.023-1.537)	0.659, <0.001	1.933 (1.381-2.705)		
Separated/Divorced/Widowed	Reference	Reference	Reference	Reference		
Education level						
Primary	-0.405, 0.083	0.667 (0.422-1.054)				
Secondary	-0.138, 0.007	0.871 (0.788-0.963)				
University	Reference	Reference				
Work status						
Unemployed	Reference	Reference	Reference	Reference	Reference	Reference
Working						
Employed	0.056, 0.519	1.058 (0.891-1.255)	0.372, 0.014	1.450 (1.080-1.947)	0.364, 0.048	1.440 (1.003-2.065)
Self-employed	-0.046, 0.678	0.955 (0.768-1.187)	0.145, 0.461	1.156 (0.787-1.698)	-0.058, 0.807	0.943 (0.591-1.505)
Civil servant	0.133, 0.168	1.142 (0.945-1.380)	0.394, 0.017	1.482 (1.073-2.047)	0.232, 0.269	1.261 (0.836-1.901)
Retired	-0.540, 0.003	0.583 (0.409-0.830)	-0.252, 0.403	0.777 (0.430-1.404)	-0.456, 0.157	0.634 (0.337-1.192)
Student/Housewife	-0.083, 0.403	0.920 (0.758-1.118)	-0.093, 0.600	0.912 (0.645-1.288)	0.066, 0.743	1.068 (0.720-1.586)
Other	-0.241, 0.089	0.786 (0.595-1.037)	0.303, 0.251	1.354 (0.807-2.270)	0.379, 0.182	1.461 (0.837-2.549)

Living situation						
Alone	Reference	Reference			Reference	Reference
Two people	0.123, 0.156	1.131 (0.954-1.341)			0.424, 0.016	1.528 (1.084-2.155)
Three to five	0.322, <0.001	1.380 (1.163-1.637)			0.692, <0.001	1.998 (1.426-2.798)
More than five	0.498, 0.005	1.645 (1.163-2.327)			-0.058, 0.896	0.943 (0.392-2.271)
Elderly in your charge						
No					Reference	Reference
One					0.046, 0.824	1.047 (0.698-1.570)
Two					0.955, 0.005	2.599 (1.344-5.027)
More than two					-0.245, 0.741	0.782 (0.183-3.346)
Able to enjoy free time						
No	Reference	Reference	Reference	Reference	Reference	Reference
Yes	-0.291, <0.001	0.275 (0.230-0.329)	-1.535, <0.001	0.215 (0.149-0.313)	-1.294, <0.001	0.274 (0.188-0.399)
March day that responded to the survey						
19					Reference	Reference
20					0.020, 0.0904	1.021 (0.733-1.421)
21					-0.011, 0.958	0.989 (0.647-1.511)
22					0.063, 0.780	1.065 (0.684-1.660)
23					0.221, 0.270	1.247 (0.842-1.846)
24					-0.141, 0.620	0.868 (0.497-1.517)
25					0.906, <0.001	2.476 (1.488-4.118)
26					0.174, 0.270	1.190 (0.874-1.620)
**Somatic disease and COVID-19 variables**						
Family/Friends infected by COVID-19						
No	Reference	Reference				

Yes	0.152, 0.004	1.164 (1.049-1.293)				
**Psychological variables**						
DASS-21 Depression						
No	Reference	Reference	Reference	Reference	Reference	Reference
Yes	1.232, <0.001	3.429 (3.129-3.757)	1.061, <0.001	2.890 (2.429-3.440)	0.960, <0.001	2.611 (2.052-3.322)
DASS-21 Anxiety						
No	Reference	Reference	Reference	Reference	Reference	Reference
Yes	1.986, <0.001	7.286 (5.907-8.988)	1.725, <0.001	5.612 (4.153-7.583)	1.920, <0.001	6.821 (4.994-9.317)
IES Intrusion						
No	Reference	Reference	Reference	Reference	Reference	Reference
Yes	1.284, <0.001	3.611 (3.254-4.007)	1.107, <0.001	3.024 (2.502-3.656)	1.296, <0.001	3.654 (2.870-4.652)
IES Avoidance						
No	Reference	Reference	Reference	Reference	Reference	Reference
Yes	0.673, <0.001	1.961 (1.790-2.147)	0.711, <0.001	2.037 (1.716-2.417)	0.443, <0.001	1.557 (1.245-1.948)
Model Chi-Square [df], p	4917.092 [21], <0.001		1333.127 [14], <0.001		1065.217 [26], <0.001	
Cox and Snell's R^2	0.279		0.305		0.349	
Nagelkerke's R^2	0.405		0.419		0.483	
H&L[1] Chi-Square [df], p	38.215 [8], <0.001		10.011 [8], 0.264		13.350 [8], 0.100	
Correct predictions	80.9%		78.0%		79.6%	

1. Hosmer and Lemeshow test.

O.R.: Odds ratio. CI: Confidence interval. df: degrees of freedom

DASS-21: Depression, Anxiety and Stress Scale (No: includes No and Doubtful; Yes: includes Mild, Moderate, Severe, and Extremely severe). IES: Impact of Event Scale.

Table S4. Intrusive thoughts response according to the IES Intrussion subscale: Variables retained in the Logistic Regression model in each of the three groups studied.

	Never Mental Disorder group (NMD) N = 14851		Past Mental Disorder group (PMD) N = 3660		Current Mental Disorder group (CMD) N = 2179	
	B, p	O.R. (95% CI)	B, p	O.R. (95% CI)	B, p	O.R. (95% CI)
**Sociodemographic variables**						
Age	0.006, 0.017	1.006 (1.001-1.012)	0.011, 0.011	1.011 (1.003-1.020)		
Gender						
Male	Reference	Reference	Reference	Reference	Reference	Reference
Female	0.315, <0.001	1.371 (1.224-1.535)	0.248, 0.027	1.281 (1.029-1.595)	0.142, 0.317	1.152 (0.873-1.522)
Civil status						
Never married			0.052, 0.777	1.054 (0.734-1.512)		
Married/Living as married			0.422, 0.010	1.524 (1.104-2.105)		
Separated/Divorced/Widowed			Reference	Reference		
Education level						
Primary					-0.80, 0.833	0.923 (0.440-1.937)
Secondary					0.075, 0.516	1.078 (0.860-1.350)
University					Reference	Reference
Work status						
Unemployed	Reference	Reference			Reference	Reference
Working						
Employed	-0.126, 0.275	0.882 (0.703-1.106)			-0.192, 0.356	0.825 (0.549-1.241)
Self-employed	-0.098, 0.495	0.907 (0.685-1.201)			0.306, 0.245	1.358 (0.811-2.275)
Civil servant	0.139, 0.269	1.149 (0.898-1.472)			0.027, 0.913	1.028 (0.630-1.676)
Retired	0.011, 0.951	1.011 (0.722-1.415)			0.010, 0.977	1.011 (0.494-2.067)

Student/Housewife	-0.317, 0.007	0.729 (0.579-0.917)	-0.409, 0.029	0.664 (0.460-0.960)
Other	0.010, 0.949	1.010 (0.747-1.364)	-0.122, 0.663	0.885 (0.511-1.532)
Income				
No income	Reference	Reference	Reference	Reference
Less than 500€	-0.118, 0.345	0.889 (0.696-1.135)	0.240, 0.239	1.272 (0.852-1.898)
500-999€	-0.075, 0.566	0.928 (0.719-1.198)	0.132, 0.546	1.141 (0.744-1.750)
1000-1499€	-0.089, 0.497	0.915 (0.707-1.183)	-0.162, 0.492	0.850 (0.536-1.350)
1500-1999€	0.116, 0.390	1.123 (0.862-1.462)	0.287,0.263	1.332 (0.806-2.201)
More than 1999€	0.010, 0.942	1.010 (0.771-1.323)	-0.008, 0.976	0.992 (0.584-1.686)
Refuse to answer	-0.217, 0.125	0.805 (0.609-1.062)	0.313, 0.223	1.368 (0.827-2.262)
Change in work status due to COVID-19				
No	Reference	Reference		
ETLA/EPLO	0.090, 0.382	1.095 (0.894-1.341)		
Dismissal	-0.322, 0.118	0.725 (0.484-1.085)		
Forced vacation	0.196, 0.108	1.216 (0.958-1.544)		
Change in income due to COVID-19				
No	Reference	Reference	Reference	Reference
Reduction, up to 25%	0.249, 0.007	1.283 (1.071-1.537)	0.173, 0.328	1.189 (0.841-1.682)
Reduction, 26-50%	0.211, 0.071	1.234 (0.982 -1.551)	-0.016, 0.938	0.984 (0.659-1.471)
Reduction, 51-100%	0.323, 0.008	1.381 (1.089-1.750)	0.002, 0.991	1.002 (0.684-1.470)
Increase	0.459, 0.104	1.582 (0.909-2.753)	0.220, 0.704	1.246 (0.400-3.882)
Living situation				
Alone	Reference	Reference		
Two people	-0.016, 0.852	0.984 (0.834-1.162)		
Three to five	-0.069, 0.448	0.933 (0.780-1.116)		
More than five	-0.579, 0.009	0.560 (0.363-0.865)		
Children in your charge				

No	Reference	Reference			Reference	Reference
One	0.174, 0.025	1.191 (1.022-1.387)			0.519, 0.001	1.680 (1.239-2.279)
Two	0.247, 0.004	1.280 (1.084-1.512)			0.110, 0.524	1.116 (0.796-1.566)
More than two	0.132, 0.403	1.141 (0.838-1.554)			0.829, 0.038	2.290 (1.047-5.009)
Able to enjoy free time						
No	Reference	Reference	Reference	Reference	Reference	Reference
Yes	-0.804, <0.001	0.448 (0.379-0.529)	-0.702, <0.001	0.496 (0.366-0.672)	-0.541, <0.001	0.582 (0.447-0.759)
March day that responded to the survey						
19	Reference	Reference	Reference	Reference		
20	-0.051, 0.507	0.950 (0.816-1.105)	0.182, 0.192	1.200 (0.913-1.577)		
21	0.112, 0.262	1.118 (0.920-1.360)	0.063, 0.727	1.065 (0.747-1.518)		
22	0.267, 0.009	1.306 (1.069-1.596)	0.434, 0.015	1.543 (1.088-2.188)		
23	0.262, 0.006	1.300 (1.079-1.566)	0.586, <0.001	1.797 (1.305-2.474)		
24	0.129, 0.391	1.137 (0.848-1.525)	0.187, 0.471	1.205 (0.725-2.002)		
25	0.358, 0.001	1.431 (1.160-1.765)	0.336, 0.082	1.393 (0.958-2.045)		
26	0.078, 0.261	1.081 (0.944-1.239)	0.122, 0.329	1.130 (0.884-1.444)		
**Somatic disease and COVID-19 variables**						
Current somatic disease*						
No					Reference	Reference
Yes					0.215, 0.043	1.240 (1.007-1.528)
Days with COVID-19 symptoms						
None					Reference	Reference
One-two days					0.045, 0.814	1.046 (0.718-1.524)
Three to five					-0.173, 0.531	0.841 (0.490-1.445)
Six to fourteen					0.418, 0.142	1.519 (0.869-2.655)
More than fourteen					0.806, 0.166	2.239 (0.715-7.015)

Family/Friends infected by COVID-19						
No	Reference	Reference				
Yes	0.147, 0.012	1.158 (1.033-1.298)				
**Psychological variables**						
DASS-21 Depression						
No	Reference	Reference	Reference	Reference	Reference	Reference
Yes	0.818, <0.001	2.266 (2.041-2.515)	0.736, <0.001	2.088 (1.728-2.524)	0.426, 0.001	1.531 (1.185-1.977)
DASS-21 Anxiety						
No	Reference	Reference	Reference	Reference	Reference	Reference
Yes	1.190, <0.001	3.286 (2.785-3.877)	0.923, <0.001	2.517 (1.975-3.208)	1.251, <0.001	3.287 (2.626-4.114)
DASS-21 Stress						
No	Reference	Reference	Reference	Reference	Reference	Reference
Yes	1.288, <0.001	3.626 (3.264-4.028)	1.154, <0.001	3.170 (2.621-3.832)	1.251, <0.001	3.492 (2.708-4.504)
IES Avoidance						
No	Reference	Reference	Reference	Reference	Reference	Reference
Yes	0.904, <0.001	2.468 (2.233-2.728)	0.889, <0.001	2.433 (2.028-2.919)	0.844, <0.001	2.325 (1.842-2.935)
Model Chi-Square [df], p	3424.186 [40], <0.001		871.111 [16], <0.001		699.930 [32], <0.001	
Cox and Snell's R^2	0.206		0.212		0.275	
Nagelkerke's R^2	0.330		0.311		0.367	
H&L[1] Chi-Square [df], p	34.093 [8], <0.001		13.233 [8], 0.104		12.938 [8], 0.114	
Correct predictions	83.6%		79.6%		74.8%	

1. Hosmer and Lemeshow test.

O.R.: Odds ratio. CI: Confidence interval. df: degrees of freedom

# ETLA: Employee Temporary Lay Off. EPLO: Employee Permanent Lay Off.

* Somatic disease includes: Hypertension, diabetes, cardiovascular diseases, respiratory diseases (asthma, chronic obstructive pulmonary disease, etc), and cancer.

DASS-21: Depression, Anxiety and Stress Scale (No: includes No and Doubtful; Yes: includes Mild, Moderate, Severe, and Extremely severe). IES: Impact of Event Scale.

Table S5. Avoidance coping style according to the IES Avoidance subscale: Variables retained in the Logistic Regression model in each of the three groups studied.

	**Never Mental Disorder group (NMD) N = 15030**		**Past Mental Disorder group (PMD) N = 3660**		**Current Mental Disorder group (CMD) N = 2489**	
	B, p	O.R. (95% CI)	B, p	O.R. (95% CI)	B, p	O.R. (95% CI)
**Sociodemographic variables**						
Age			-0.008, 0.008	0.992 (0.986-0.998)	-0.014, 0.001	0.986 (0.978-0.994)
Gender						
Male	Reference	Reference	Reference	Reference	Reference	Reference
Female	0.549, <0.001	1.732 (1.598-1.878)	0.562, <0.001	1.754 (1.471-2.091)	0.631, <0.001	1.879 (1.494-2.363)
Education level						
Primary	0.507, 0.001	1.660 (1.228-2.245)	0.492, 0.113	1.635 (0.890-3.004)	0.372, 0.229	1.451 (0.791-2.660)
Secondary	0.231, <0.001	1.260 (1.159-1.369)	0.382, <0.001	1.466 (1.257-1.709)	0.282, 0.004	1.326 (1.094-1.606)
University	Reference	Reference	Reference	Reference	Reference	Reference
Income (€)						
No income	Reference	Reference				
Less than 500	0.000, 0.998	1.000 (0.847-1.181)				
500-999	-0.157, 0.025	0.854 (0.744-0.981)				
1000-1499	-0.198, 0.002	0.820 (0.724-0.928)				
1500-1999	-0.263, <0.001	0.769 (0.674-0.876)				
More than 1999	-0.444, <0.001	0.641 (0.560-0.734)				
Refuse to answer	-0.095, 0.273	0.909 (0.767-1.078)				
Living situation						
Alone					Reference	Reference
Two people					-0.083, 0.583	0.920 (0.685-1.237)

Three to five					0.225, 0.159	1.252 (0.916-1.713)
More than five					-0.165, 0.684	0.848 (0.382-1.879)
Children in your charge						
No	Reference	Reference			Reference	Reference
One	-0.165, 0.002	0.848 (0.764-0.941)			-0.063, 0.675	0.939 (0.701-1.259)
Two	-0.281, <0.001	0.755 (0.678-0.841)			-0.365, 0.033	0.694 (0.496-0.971)
More than two	-0.366, 0.001	0.694 (0.554-0.868)			-0.568, 0.103	0.566 (0.286-1.123)
Able to enjoy free time						
No	Reference	Reference	Reference	Reference		
Yes	-0.424, <0.001	0.655 (0.557-0.770)	-0.307, 0.062	0.736 (0.533-1.016)		
March day that responded to the survey						
19	Reference	Reference	Reference	Reference		
20	0.124, 0.026	1.132 (1.015-1263)	0.069, 0.541	1.071 (0.859-1.335)		
21	0.042, 0.575	1.043 (0.900-1.210)	-0.071, 0.629	0.932 (0.699-1.242)		
22	0.181, 0.022	1.198 (1.026-1.398)	0.342, 0.030	1.408 (1.033-1.919)		
23	0.132, 0.076	1.141 (0.986-1.319)	0.283, 0.051	1.327 (0.999-1.763)		
24	0.263, 0.022	1.301 (1.039-1.629)	0.212, 0.335	1.237 (0.803-1.904)		
25	-0.013, 0.0876	0.987 (0.833-1.168)	0.061, 0.710	1.062 (0.771-1.463)		
26	0.113, 0.029	1.119 (1.012-1.238)	0.297, 0.004	1.346 (1.099-1.649)		
**Psychological variables**						
DASS-21 Depression						
No	Reference	Reference	Reference	Reference	Reference	Reference
Yes	0.588, <0.001	1.801 (1.666-1.946)	0.380, <0.001	1.462 (1.256-1.703)	0.420, <0.001	1.522 (1.230-1.885)
DASS-21 Anxiety						
No	Reference	Reference	Reference	Reference	Reference	Reference
Yes	0.402, <0.001	1.495 (1.262-1.771)	0.648, <0.001	1.911 (1.456-2.509)	0.612, <0.001	1.844 (1.452-2.342)
DASS-21 Stress						

No	Reference	Reference	Reference	Reference	Reference	Reference
Yes	0.677, <0.001	1.969 (1.800-2.154)	0.717, <0.001	2.048 (1.725-2.431)	0.484, <0.001	1.623 (1.305-2.019)
IES Intrusion						
No	Reference	Reference	Reference	Reference	Referenc	Reference
Yes	0.860, <0.001	2.364 (2.141-2.160)	0.864, <0.001	2.374 (1.978-2.848)	0.880, <0.001	2.411 (1.942-2.992)
Model Chi-Square [df], p	2633.724 [24], <0.001		676.227 [17], <0.001		460.213 [14], <0.001	
Cox and Snell's R^2	0.161		0.169		0.169	
Nagelkerke's R^2	0.218		0.225		0.236	
H&L[1] Chi-Square [df], p	21.225 [8], 0.007		12.248 [8], 0.188		1.198 [8], 0.997	
Correct predictions	70.2%		66.7%		73.4%	

1. Hosmer and Lemeshow test.

O.R.: Odds ratio. CI: Confidence interval. df: degrees of freedom

DASS-21: Depression, Anxiety and Stress Scale (No: includes No and Doubtful; Yes: includes Mild, Moderate, Severe, and Extremely severe). IES: Impact of Event Scale.
